# Tumor cells utilize acetate for tumor growth and immune evasion

**DOI:** 10.1002/mco2.717

**Published:** 2024-08-31

**Authors:** Peng Sun, Juanjuan Liu, Deliang Guo

**Affiliations:** ^1^ Departments of Hepatobiliary and Pancreatic Surgery and Oncology The Affiliated Hospital of Qingdao University Qingdao Cancer Institute Qingdao China; ^2^ Department of Radiation Oncology Center for Cancer Metabolism Ohio State Comprehensive Cancer Center Arthur G. James Cancer Hospital and Richard J. Solove Research Institute, and College of Medicine at The Ohio State University Columbus Ohio USA

1

A recent study from Zhimin Lu's group published in *Nature Metabolism* ^1^ demonstrates that acetate reprogrammed cancer cell metabolism and promoted tumor immune evasion. Notably, nutrients, such as glucose and short‐chain fatty acids (SCFAs), in the tumor microenvironment impact tumor growth.^1^ It is well known that tumor cells, regardless of oxygen supply, utilize glucose to produce ATP and building blocks for macromolecule synthesis^2^, ^3^; however, recent research has shown that glucose uptake by these cells not only supports the Warburg effect but also triggers non‐metabolic functions.^4^ Similar to glucose as a nutrient derived from the diet, acetate, as a main SCFA, is also enriched in the tumor microenvironment. Acetate plays a critical role in mitochondrial oxidation, lipogenesis, and histone acetylation to support tumor cell growth.^5^ However, it remains unclear whether acetate contributes to tumor cell proliferation and immune evasion by directly influencing oncogenic proteins.

Through metabolomic analysis of human non‐small cell lung cancer (NSCLC) specimens, Lu's team revealed that acetate was the most abundant short‐chain fatty acid (SCFA). They found that the carbon‐13 (^13^C)‐ or deuterium (^3^D)‐labeled acetate was more enriched in lung tumor tissues and tumor cells than in normal lung tissues and tumor interstitial fluid in mice, with a corresponding increase in ^13^C‐acetyl‐CoA in tumor tissues.^1^ Depletion studies of monocarboxylate transporters (MCT)1‐4 and sodium‐coupled MCT (SMCT)1‐2 showed that only the depletion of MCT1, which is highly expressed in NSCLC tissues, led to reduced levels of acetate, acetyl‐CoA, and synthesized fatty acids in tumor cells or mouse lung tumors. These results indicate that highly expressed MCT1 transports acetate into tumor cells. Notably, acetate supported tumor cell proliferation and mouse tumor growth only under conditions of low glucose or depletion of glucose transporters GLUT1 and GLUT3,^1^ suggesting that glucose is a primary resource for tumor growth and that acetate counteracts energy stress to sustain tumor cell proliferation.

In addition to supporting anabolic synthesis, acetate‐derived acetyl‐CoA, produced by the enzyme acetyl‐CoA synthetase 2 (ACSS2), is utilized for protein acetylation. Mass spectrometry analysis of cellular immunoprecipitates with an anti‐acetylated lysine antibody showed that acetate increased both the acetylation and expression of c‐Myc. In addition, acetate increased the interaction between c‐Myc and dihydrolipoamide S‐acetyltransferase (DLAT), a component of pyruvate dehydrogenase complex (PDC).^1^ Remarkably, purified DLAT was able to acetylate purified c‐Myc at the K148 site in vitro. In NSCLC cells, depletion of DLAT reduced the acetylation and expression of c‐Myc at K148, while increasing c‐Myc polyubiquitylation. Consistently, acetylation‐dead c‐Myc K148R and acetylation‐mimicking c‐Myc K148Q mutations decreased and increased c‐Myc half‐life, respectively.^1^ These results indicate that DLAT acts as a bona fide protein acetyltransferase to acetylate and stabilize c‐Myc.

Through mass spectrometry analyses, ubiquitin‐specific peptidase 10 (USP10) was identified as a c‐Myc‐associated protein and deubiquitylated c‐Myc.^1^ A GST pulldown assay demonstrated that DLAT‐mediated c‐Myc acetylation facilitated the binding of wild‐type (WT) c‐Myc, but not the c‐Myc K148R mutant, to USP10. Acetate supplementation increased the binding of USP10 to c‐Myc but not c‐Myc K148R in a DLAT expression‐dependent manner whereas c‐Myc K148Q increased its association with USP10 compared to its WT counterpart.^1^ Additionally, depletion of USP10 increased c‐Myc polyubiquitylation and degradation, and these effects were not reversed by reconstituted USP10 expression when DLAT was depleted. These results indicate that acetate‐enhanced and DLAT‐mediated c‐Myc K148 acetylation induces the binding of USP10 to c‐Myc, leading to c‐Myc deubiquitylation and stabilization.

c‐Myc activation is known to induce expression of *CD274* (encoding PD‐L1), *CCND1* (encoding cyclin D1), *LDHA* (encoding lactate dehydrogenase A), and *MCT1*.^1^ As expected, acetate supplementation under low‐glucose conditions enhanced the expression of PD‐L1, cyclin D1, LDHA, and MCT1, MCT1‐dependent acetate uptake, lactate production, and tumor cell proliferation.^1^ In addition, coculture of ovalbumin (OVA)‐expressing mouse lung cancer cells with mouse CD8^+^ T cells expressing a transgenic T cell receptor (TCR) specific for an ovalbumin peptide showed that the acetate‐treated tumor cells inhibited the expression of interleukin‐2 and interferon‐γ in CD8^+^ T cells. These acetate‐induced effects were abolished by the depletion of MCT1, ACSS2, DLAT, and USP10, or by the knockin expression of the c‐Myc K148R mutant.^1^ These results indicate that acetate facilitates acetate uptake, glycolysis, and NSCLC cell proliferation, and PD‐L1‐dependent inhibition of CD8^+^ T‐cell activation dependent on the acetate‐MCT1‐ACSS2‐DLAT‐USP10‐c‐Myc axis (Figure 1).

**FIGURE 1:**
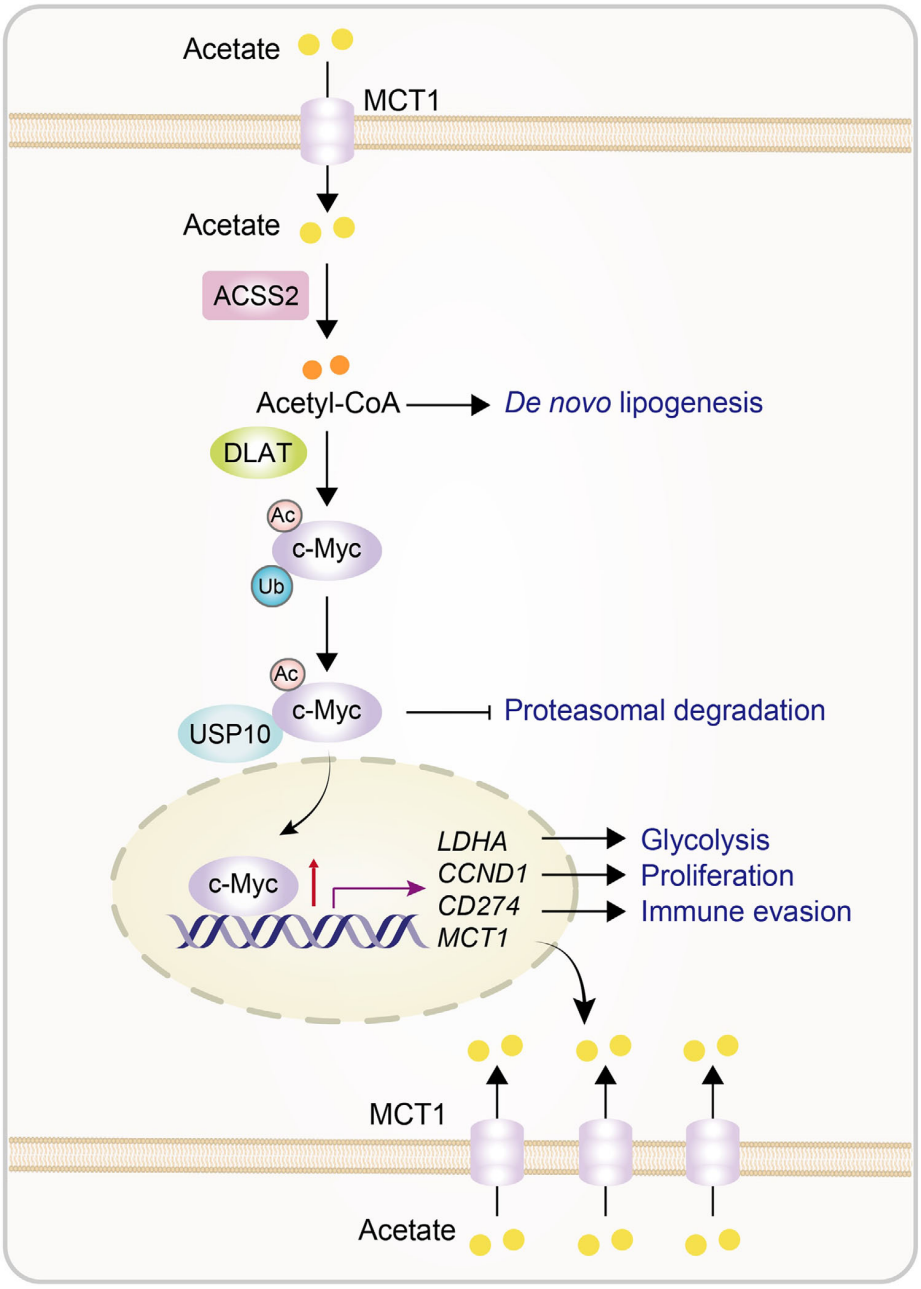
**Acetate promotes tumor growth and immune evasion**. Acetate promotes tumor growth and immune evasion through the MCT1‐ACSS2‐DLAT‐USP10‐c‐Myc signaling axis. In tumor cells, MCT1‐transported acetate is converted to acetyl‐CoA by ACSS2. Acetyl‐CoA is utilized not only for de novo lipogenesis but also for c‐Myc acetylation by DALT. Acetylated c‐Myc recruits USP10 for deubiquitination and stabilization of c‐Myc. This enhances the expression of LDHA, cyclin D1, and PD‐L1, leading to increased glycolysis, tumor cell proliferation, and tumor immune evasion, respectively. c‐Myc also upregulates MCT1, thereby forming a positive feedback loop for acetate uptake. ACSS2: acetyl‐CoA synthetase 2; DLAT: dihydrolipoamide S‐acetyltransferase; LDHA: lactate dehydrogenase A; MCT1: monocarboxylate transporter 1; USP10: ubiquitin specific peptidase 10.

Analyses of single‐cell sequencing datasets from NSCLC and small‐cell lung cancer tissues showed that *MCT1* mRNA levels in tumor cells were higher than those in tumor‐infiltrating lymphocytes (TILs).^1^ ^13^C_2_‐acetate isotope‐tracing experiments showed that murine lung cancer cells exhibited much higher acetate uptake than tumor‐infiltrating CD45^+^ leukocytes in mouse lung, suggesting that acetate is more efficiently taken up by tumor cells than TILs.^1^ Time‐of‐flight mass cytometry (CyTOF) analysis of mouse tumors revealed that acetate supplementation in drinking water decreased the infiltration of cytotoxic CD8^+^ T cells, CD4^+^ T helper 1 (Th1) cells, and M1 macrophages and increased the infiltration of CD4^+^ (Th2) cells and myeloid‐derived suppressor cells (MDSCs) in mouse tumors. Acetate also elevated the levels of pro‐tumor cytokines, chemokines, and growth factors while decreasing the production of anti‐tumor cytokines and factors. This was accompanied by increased tumor tissue expression of c‐Myc, Ki‐67, MCT1, LDHA, and PD‐L1, as well as decreased CD8^+^ T‐cell infiltration and granzyme B expression. These changes promoted tumor growth and shortened mouse survival time in the presence of glycolysis inhibitor treatment. Reconstituted expression of c‐Myc K148R, depletion of MCT1, ACSS2, DLAT and USP10, or treatment with the USP10 inhibitor spautin‐1 diminished acetate‐induced effect in mice.^1^ Notably, combined treatment with spautin‐1 and an anti‐PD‐1 antibody had an additive effect. These findings indicate that acetate‐mediated acetylation of c‐Myc at K148 in tumor cells creates an immunosuppressive tumor microenvironment and promotes tumor growth. Inhibition of this pathway eliminates the acetate‐induced effect and enhances the efficacy of immune checkpoint blockade therapy. Analyses of 90 human NSCLC tissues showed that c‐Myc K148 acetylation levels were positively associated with the MCT1, c‐Myc, and PD‐L1 expression levels and inversely correlated with CD8^+^ T cell infiltration. In addition, c‐Myc K148 acetylation or USP10 expression levels were associated with poor survival of the patients.

Tumor heterogeneity is evident in the differing levels of GLUT1 expression within tumor tissues. This study showed that tumor cells with low GLUT expression or glucose uptake can use acetate as a primary source for acetyl‐CoA production and lipid biosynthesis. Acetate also induces c‐Myc‐dependent MCT1 expression at the transcriptional level. As a result, acetate uptake is further amplified by the acetate‐c‐Myc‐MCT1 positive feedback loop in tumor cells. Importantly, in addition to its role as a metabolic carbon source, acetate reprogrammed tumor cell metabolism and promoted immune evasion through posttranslational modification of c‐Myc, which depends on the moonlighting protein acetyltransferase activity of DLAT. Thus, these findings underscore not only the potential of using labeled acetate for cancer diagnosis and monitoring tumor growth but also highlight the possibility of targeting the MCT1‐ACSS2‐DLAT‐USP10‐c‐Myc signaling axis for cancer treatment, including enhancing the response to immune checkpoint blockade therapy. Importantly, the finding that acetate promotes tumor growth through tumor cell‐intrinsic signaling and tumor immune evasion through the regulation of immune checkpoints highlights the significant impact of dietary components on cancer progression, revealing the potential of dietary‐based therapy for cancer treatment.

## AUTHOR CONTRIBUTIONS

Deliang Guo, Peng Sun, and Juanjuan Liu conceptualized the writing. All authors together wrote the manuscript. All authors have read and approved the article.

## CONFLICT OF INTEREST STATEMENT

The authors declare no conflict of interest.

## ETHICS STATEMENT

N/A

## Data Availability

N/A
